# Supplementary figures and images for: Relevance of the bacteriophage adherence to mucus model for Pseudomonas aeruginosa phages
Source: Microbiol Spectr. 2024 Jun 24;12(8):e03520-23. doi: 10.1128/spectrum.03520-23 (PMC11302309; doi:10.1128/spectrum.03520-23)

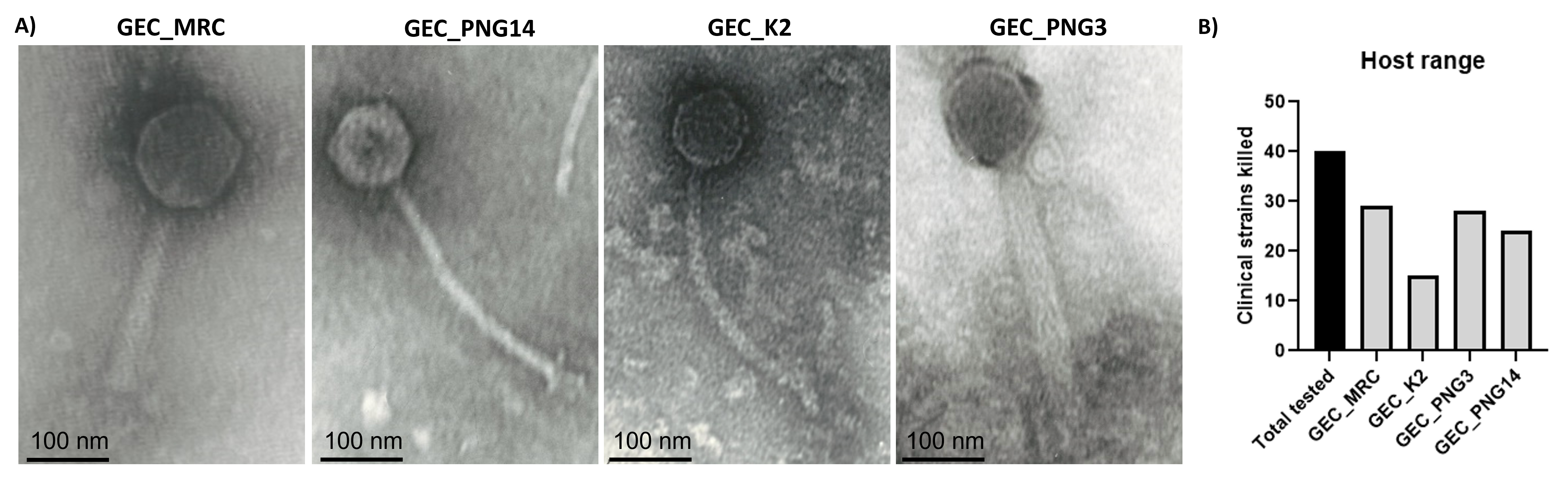

Supplement: Figure S1 — Imaging and host range of the phages used in this study. [file spectrum.03520-23-s0001.tif]

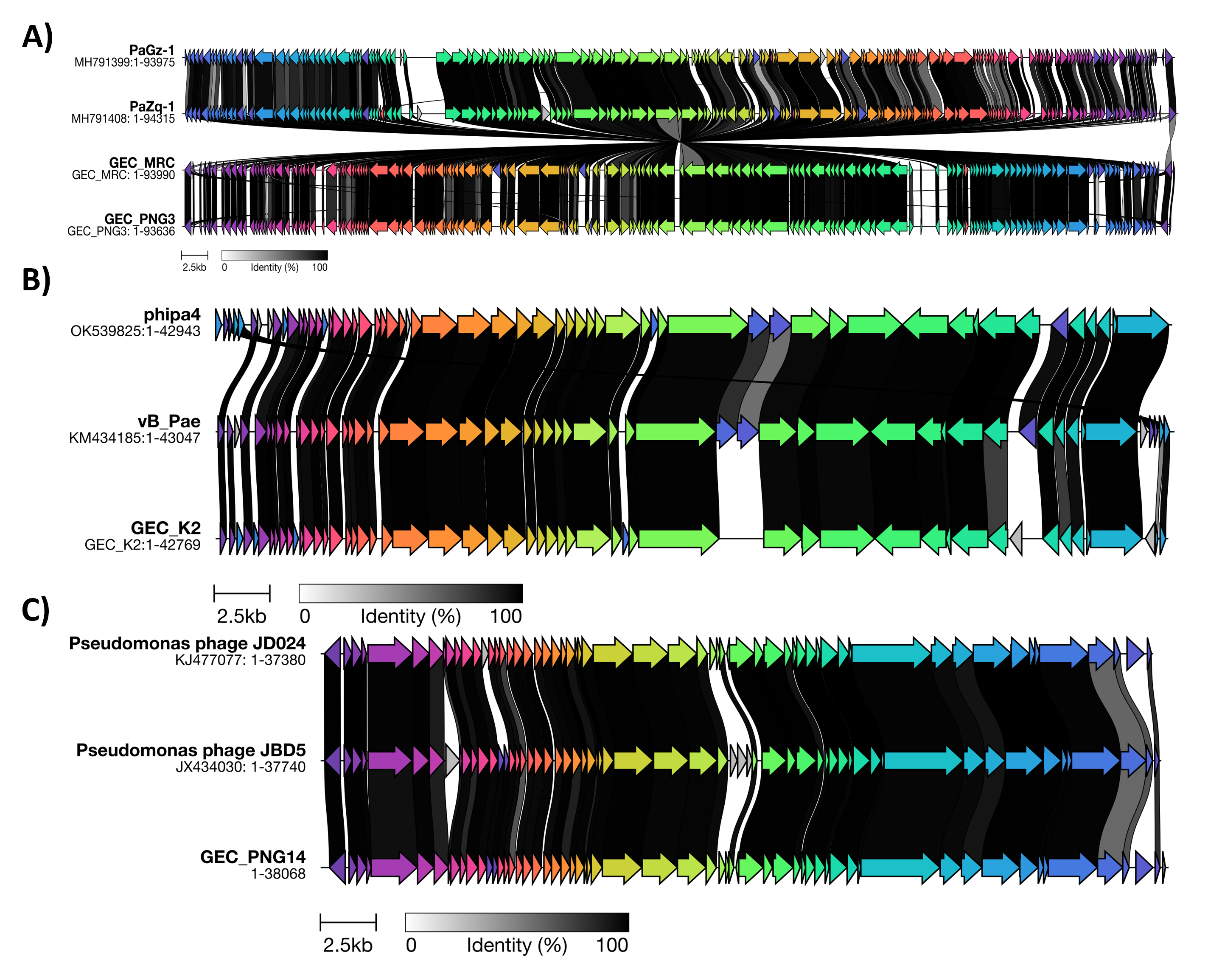

Supplement: Figure S2 — Genomic organization and comparison of Pseudomonas aeruginosa phages. [file spectrum.03520-23-s0002.tif]
