# Supplementary material for: Relevance of the bacteriophage adherence to mucus model for Pseudomonas aeruginosa phages
Source: Microbiol Spectr. 2024 Jun 24;12(8):e03520-23. doi: 10.1128/spectrum.03520-23 (PMC11302309; doi:10.1128/spectrum.03520-23)
Supplement: Supplemental text — Characterization of the phages, host used in this study, and Tables S1 and S2. [file spectrum.03520-23-s0003.docx]

# **Supplementary text.** Characterization of the phages and host used in this study.

Phage morphology is shown in **supplementary figure 1A**. When screened against 40 clinical strains of *P. aeruginosa* isolated in Georgia from the sputum, ear, nose, pharynx, tooth, nose and urine samples of patients, host range varied between the phages (**supplementary figure 1B**). The best activity was demonstrated by the GEC_MRC and GEC_PNG3 phages (myovirus) that lysed 72,5% and 70% of the tested strains, respectively. The siphovirus phages showed weaker activity to the clinical strains, varying from 60% (GEC_PNG14) to 37% (GEC_K2). Of the four phages, only GEC_PNG14 was able to grow in the reference *P. aeruginosa* strain PA14. Additional details about the phages are shown in the **supplementary table 1**.

**Supplementary table 1**: Isolation and morphological details of phages used in this study.

| **Phage** | **Host strain** | **Isolation date** | **Phage source** | **Plaque morphology** | **Virion morphology type** |
| --- | --- | --- | --- | --- | --- |
| GEC_MRC | P.a 573 | 2015 | r. Mtkvari, Tbilisi, Georgia | Clear centre Ø4mm, surrounded by the turbid concentric circles of the halo 3mm | myovirus |
| GEC_PNG3 | P.a 573 | 2018 | Waste water, Tbilisi, Georgia | Clear centre Ø5mm, surrounded by the turbid halo 3mm | myovirus |
| GEC_K2 | P.a 573 | 2018 | r. Mtkvari, Tbilisi, Georgia | Clear plaques  Ø 0.3mm | siphovirus |
| GEC_PNG14 | P.a 573 | 2019 | Waste water, Tbilisi, Georgia | Clear centre Ø 0.4mm surrounded by the turbid halo 2mm | siphovirus |

**Genomic analysis of *P. aeruginosa* phages**

All the four phages used in this study were sequenced. De novo assembly of the four *P. aeruginosa* phage genomes produced assemblies of varying lengths >93 kbp for GEC_MRC and GEC_PNG3, 42,8 kbp for GEC_K2 and 37,6 kbp for GEC_PNG14. Main results of genome assemblies are included in **supplementary table 2**. **Supplementary tables** 3-6 shows the BLASTp annotations for each phage.

**Supplementary table 2**: Characteristics of phage genome assemblies. **NOTE FOR THE EDITOR AND REVIEWERS**: genome accession numbers will be added. For now the genomes can be accessed for review purposes at the link provided under the “data availability” section.

| **Phage name** | **Genome length (bp)** | **GC%** | **number of tRNA genes** |
| --- | --- | --- | --- |
| GEC_PNG14 | 38 068 | 64,1 | 0 |
| GEC_K2 | 42 769 | 53,7 | 0 |
| GEC_MRC | 93 990 | 49,4 | 15 |
| GEC_PNG3 | 93 636 | 49,3 | 15 |

PhageTerm analysis (**1**) for physical phage genome ends and packaging strategy suggested *cos* packaging for phages GEC_MRC and GEC_PNG3. They were also observed to have direct terminal repeats (CCACGAACCCACCTCGCTACCCT). At nucleotide level (global alignment), GEC_MRC and GEC_PNG3 share 95.2% identity despite their different origin. At amino acid level, their ORFS are >90% similar. With these phages, the closest BLASTp hits were phages PaZq-1 and PaGz-1 (*Pakpunaviruses),* isolated from China (**2**), but the nucleotide-level identity remained below 50%. Both phages are 47.7% identical to PaZq-1, and when compared to PaGz-1 identities are 47,6% for GEC_MRC and 47,8% for GEC_PNG3 (**Figure S2**). Average nucleotide identity (ANI) shows higher identities: 95,68% between GEC_MRC and GEC_PNG3, 93,9% between GEC_MCR and PaZq1, and 94,25% between GEC_PNG3 and PaZq1. However, according to ANI, these phages more closely related to Pakpunavirus group CAb02, as GEC_MRC showed 95,11% ANI with *Pseudomonas* phage vB_PA45_GUMS (MN563785) and 95,09% with Pseudomonas phage YS35 (MF974178.1).

BLASTp annotation of GEC_MRC and GEC_PNG3 identified DNA polymerase (locus tags GEC_MRC_068 and GEC_PNG3_068, **Supplementary Tables** 3 and 4) and clusters of structural genes (putative tail fiber proteins, baseplate, tail tape measure protein) in area between 45kbp and 55 kbp in both phages. Major capsid protein was also identified in GEC_PNG3 (GEC_PNG3_114) and GEC_MRC (GEC_MRC_115), in addition to putative cell wall hydrolases (GEC_MRC_139 and GEC_PNG3_138). Also, putative functions allowing pyrimidine salvage were found: ribose-phosphate pyrophosphokinase and nicotinamide phosphoribosyltransferase (GEC_MRC_148 and GEC_MRC_150 in GEC_MRC, and GEC_PNG3_147 and GEC_PNG3_149 in PNG3, respectively). Furthermore, 15 tRNA’s were identified in each of these phages (**Supplementary table** 5). HMM-HMM comparisons (HHpred with UniRef 100 database) analysis revealed hits to Ig-binding and carbohydrate-binding domains. In both phages, two hits to Ig-binding domains were found, both being hypothetical proteins: GEC_MCR_097 (probability 96.88%, e-Value 9.4E-05), GEC_MCR_114 (96.02%, 0.0022), GEC_PNG3_096 (98,18%, 2.6E-08) and GEC_PNG3_113 (96%, 0.0022). Hits carbohydrate-binding domains were found in two putative tail fiber proteins in each phage: GEC_MRC_094 (99,5%, 9.5E-13), GEC_MRC_096 (99,5%, 9.5E-13), GEC_PNG3_093 (99,05%, 9.5E-13) and GEC_PNG3_095 (99,71%, 1.1E-20),

For phage GEC_K2 PhageTerm suggested headful (*pac*) packaging and circularly permuted genome. Glimmer predicted 61 ORFs, of which 53 were annotated. Majority of the ORFs remained hypothetical proteins in BLASTp annotation (**Supplementary table** 6), although some ORFs gave hits to structural proteins. Also a putative holin (GEC_K2_018) and two terminase-like proteins were identified (GEC_K2_021 and GEC_K2_022). DNA polymerase (GEC_K2_040) replicative clamp (GEC_K2_041) and replicative primase/helicase protein (GEC_K2_050) were identified, as well as MazG (GEC_K2_048), which may be related to host survival under starvation (**3**). HMM-HMM comparisons (HHpred with UniRef 100 database) revealed three ORFS with significant hits to Ig-binding domains: GEC_K2_019 (hypothetical protein with TIGR02594 domain (probablility 99.89%, significance 3.8E-27) GEC_K2_032 (major tail tube protein, 99.96%, 2.5E-33) and GEC_K2_039 (central tail hub protein, 99.96%, 3.1E-33). Furthermore a hypothetical protein (GEC_K2_008) had a hit to a carbohydrate-binding domain (99.61%, 1.2E-18). GEC_K2 showed high similarity to previously isolated phages phipa4 and vB_Pae (global nucleotide identity 91,2% and 95%, respectively) (**Figure** S**2B**). Average nucleotide identity of GEC_K2 with phipa4 is 97,3% and 97,5% with vB_Pae.

Physical ends could not be determined for GEC_PNG14. BLASTp revealed genetic features associated with lysogenic lifestyle (**Supplementary table** 7), although this phage (as well as the other phages K2, MRC and PNG3) are lytic in the laboratory conditions used in the described experiments. ORF1 was identified as helix-turn-helix transcriptional regulator, and ORF2 as another helix-tur-helix domain protein, and ORF5 (GEC_PNG14_005) as a transposase. ORF10 (GEC_PNG14_010) gave a hit to host-nuclease inhibitor Gam family protein, which has been suggested to provide phage Mu protection from exonucleases (**4**). In addition, a Mu-like prophage head protein (GEC_PNG14_036) was found. Additional RAST annotation (**5**) revealed even more hits to Mu-like proteins (data not shown). Bioinformatic analysis of PNG14 suggested two putative Anti-CRISPR proteins in PNG14. ORF31 (locus tag: GEC_PNG14_031) was annotated as AcrIF2 in the initial BlastP analysis, and additional HHpred analysis (using PDB database) gave a hit to Pseudomonas phage D112 anti-CRISPR protein 30 (Probability 100, E-value 1.7e-51). Interestingly, also the following ORF (locus tag: GEC_PNG14_032) had a hit to anti-CRIPSR AcrIE4 in BlastP, and also a 100% confidence hit to the same Anti-CRISPR type in Phyre (96% Alignment coverage). HMM-HMM analysis revealed an Ig-binding domain in PNG14 tail protein (GEC_PNG14_051, 99.8%, 4.4E-24) and a carbohydrate- binding domain in phage BR0599 family protein (GEC_PNG14_ 048, 99.3%, 1E-14). Phage PNG14 is similar to previously isolated *P. aeruginosa* phages JD024 and JBD5, with ANI of 98,68% and 97,7%, respectively (global nucleotide alignment identity: 50,2% and 49,9%, respectively) (**supplementary figure 2**). According to Adriaenssens et al 2015 (**6**) phages JD024 and JBD5 are temperate and related to *P. aeruginosa* phage D3112 (**7**).

**CRISPR-Cas spacers and prophages in *P. aeruginosa* strain 573.**

*P. aeruginosa* is known to possess CRISPR systems. The reference strain PA14 for example has a type I-F CRISPR-Cas system with two CRISPR regions flanking its *cas* genes, shown to be active in different conditions (**8-10**). The phages used in this study infect a different strain, *P. aeruginosa* 573. Whole genome annotation (5813 CDS) of this strain is available in *Pseudomonas* genome DB under name *Pseudomonas aeruginosa* CN573=PSE143. Here, we analysed the CRISPR-Cas spacers and prophages found in this strain. CRISPRCasFinder (**11**) identified a Type I-F CRISPR-Cas system with 39 spacers in contig 12. Perfect CRISPR spacer matches to known phage sequences and the phages used in this study are listed in **Supplementary table** 8. One spacer was found to match GEC_PNG14.

Prophage Hunter and Phaster analyses recognized the same prophage in *P. aeruginosa* 573. Eight open reading frames (ORFs) of this 18.8 kb long prophage with 23 ORFs and GC% of 66,56 had hits to *Pseudomonas* phage YMC11/02/R656, which is a siphovirus with a genome size of 61kbp (NC_028657). Four prophage ORFs were annotated as tail proteins by Phaster, and the last ORF as an excisionase. The prophage was suggested to be active by Prophage Hunter (score 0.86), but Phaster analysis identified it with an incomplete status. Prophage annotation is provided in **Supplementary table** 9.

# Supplementary figure legends

**Supplementary figure 1:** Imaging and host range of the phages used in this study. A) Transmission electron microscopy of the phages. B) Host range of each phage against 40 clinical isolates of *P. aeruginosa* obtained from patients in Georgia.

**Supplementary figure 2.** Genomic organization and comparison of *Pseudomonas aeruginosa* phages. A) GEC_MRC and GEC_PNG3 vs Pseudomonas phages PaGz1 and PaZq1, b) GEC_K2 vs *Pseudomonas* phages vB_Pae and phipa4, c) GEC_PNG14 vs *Pseudomonas* phages JD024 and JBD5. Comparisons and figures generated with clinker (**12**).

# References

1. Garneau, J.R., Depardieu, F., Fortier, LC. et al. PhageTerm: a tool for fast and accurate determination of phage termini and packaging mechanism using next-generation sequencing data. Sci Rep 7, 8292 (2017). https://doi.org/10.1038/s41598-017-07910-5

2. Wen, L., Chen, L., Yuan, S. et al. Complete genome analysis of PaGz-1 and PaZq-1, two novel phages belonging to the genus Pakpunavirus. Arch Virol 165, 2393–2396 (2020). https://doi.org/10.1007/s00705-020-04745-w

3. Clokie, M.R.J. and Mann, N.H. (2006), Marine cyanophages and light. Environmental Microbiology, 8: 2074-2082. https://doi.org/10.1111/j.1462-2920.2006.01171.x

4. Akroyd JE, Clayson E, Higgins NP. Purification of the gam gene-product of bacteriophage Mu and determination of the nucleotide sequence of the gam gene. Nucleic Acids Res. 1986 Sep 11;14(17):6901-14. doi: 10.1093/nar/14.17.6901. PMID: 2945162; PMCID: PMC311707.

5. Aziz RK, Bartels D, Best AA, et al. The RAST Server: rapid annotations using subsystems technology. BMC Genomics. 2008;9:75. Published 2008 Feb 8. doi:10.1186/1471-2164-9-75

6. Adriaenssens EM, Edwards R, Nash JHE, Mahadevan P, Seto D, Ackermann HW, Lavigne R, Kropinski AM. Integration of genomic and proteomic analyses in the classification of the Siphoviridae family. Virology. 2015 Mar;477:144-154. doi: 10.1016/j.virol.2014.10.016.

7. Wang PW, Chu L, Guttman DS. Complete sequence and evolutionary genomic analysis of the Pseudomonas aeruginosa transposable bacteriophage D3112. J Bacteriol. 2004;186(2):400-410. doi:10.1128/JB.186.2.400-410.2004

8. Høyland-Kroghsbo NM, Paczkowski J, Mukherjee S, Broniewski J, Westra E, Bondy-Denomy J, Bassler BL. Quorum sensing controls the Pseudomonas aeruginosa CRISPR-Cas adaptive immune system. Proc Natl Acad Sci U S A. 2017 Jan 3;114(1):131-135. doi: 10.1073/pnas.1617415113

9. Høyland-Kroghsbo NM, Muñoz KA, Bassler BL. Temperature, by Controlling Growth Rate, Regulates CRISPR-Cas Activity in Pseudomonas aeruginosa. mBio. 2018 Nov 13;9(6):e02184-18. doi: 10.1128/mBio.02184-18.

10. Alseth, E. O., Pursey, E., Luján, A. M., McLeod, I., Rollie, C. & Westra, E. R. 2019 Bacterial biodiversity drives the evolution of CRISPR-based phage resistance. Nature 574, 549–552. (doi:10.1038/s41586-019-1662-9)

11. Abby, S. S.,Neron, B., Menager, H., Touchon, M., Rocha, E. P. : MacSyFinder: a program to mine genomes for molecular systems with an application to CRISPR-Cas systems, PloSOne, 9: e110726, 2014.

12. Gilchrist CLM, Chooi YH. clinker & clustermap.js: automatic generation of gene cluster comparison figures. Bioinformatics. 2021 Aug 25;37(16):2473-2475. doi: 10.1093/bioinformatics/btab007. PMID: 33459763.
